# Assessing Commitment and Reporting Fidelity to a Text Message-Based Participatory Surveillance in Rural Western Uganda

**DOI:** 10.1371/journal.pone.0155971

**Published:** 2016-06-09

**Authors:** James Lester, Sarah Paige, Colin A. Chapman, Mhairi Gibson, James Holland Jones, William M. Switzer, Nelson Ting, Tony L. Goldberg, Simon D. W. Frost

**Affiliations:** 1 Department of Veterinary Medicine, University of Cambridge, Cambridge, United Kingdom; 2 Department of Pathobiological Sciences, University of Wisconsin-Madison, Madison, WI, United States of America; 3 Global Health Institute, University of Wisconsin-Madison, Madison, WI, United States of America; 4 Department of Anthropology and McGill School of Environment, McGill University, Montreal, Canada; and Wildlife Conservation Society, Bronx, New York, United States of America; 5 Department of Archaeology and Anthropology, University of Bristol, Bristol, United Kingdom; 6 Department of Anthropology, Woods Institute for the Environment, Stanford University, Stanford, CA, United States of America; 7 Laboratory Branch, Division of HIV/AIDS Prevention, National Center for HIV, Hepatitis, STD and TB Prevention, Centers for Disease Control and Prevention, Atlanta, GA, United States of America; 8 Department of Anthropology, University of Oregon, Eugene, OR, United States of America; University of South Africa (South Africa) and University of Tennessee (USA), UNITED STATES

## Abstract

Syndromic surveillance, the collection of symptom data from individuals prior to or in the absence of diagnosis, is used throughout the developed world to provide rapid indications of outbreaks and unusual patterns of disease. However, the low cost of syndromic surveillance also makes it highly attractive for the developing world. We present a case study of electronic participatory syndromic surveillance, using participant-mobile phones in a rural region of Western Uganda, which has a high infectious disease burden, and frequent local and regional outbreaks. Our platform uses text messages to encode a suite of symptoms, their associated durations, and household disease burden, and we explore the ability of participants to correctly encode their symptoms, with an average of 75.2% of symptom reports correctly formatted between the second and 11th reporting timeslots. Concomitantly we identify divisions between participants able to rapidly adjust to this unusually participatory style of data collection, and those few for whom the study proved more challenging. We then perform analyses of the resulting syndromic time series, examining the clustering of symptoms by time and household to identify patterns such as a tendency towards the within-household sharing of respiratory illness.

## Introduction

The core concept of using the observation of a given symptom, or combination of symptoms (i.e. a syndrome) to understand the pattern of occurrence of a given disease is arguably one of the oldest forms of disease surveillance, however only relatively recently has syndromic surveillance become a formal part of public health surveillance in a number of developed nations. Syndromic surveillance has been defined by the U.S. Centers for Disease Control and Prevention (CDC) as “a process that regularly and systematically uses health and health-related data in near “real-time” to make information available on the health of a community” [1]. Syndromic surveillance can take a wide range of forms, from the screening of electronic health records, to the collection of pre-diagnostic symptom data in a healthcare or community setting, to the monitoring of pharmaceutical purchasing behaviour. Syndromic surveillance has many useful qualities for disease surveillance; it does not require a final diagnosis from a medical professional and thus can provide very early indications of unusual patterns of disease; it can take advantage of sophisticated technology, whilst also being amenable to ‘low technology’ approaches [2]; it can monitor novel illnesses lacking clear case definitions; and it can operate within a healthcare or a community setting. Syndromic surveillance is an exceedingly flexible tool, capable of being implemented in many different contexts, and modified to best suit a given goal. This is reflected by the wide range of modern surveillance efforts using it.

Different types of surveillance systems, and therein syndromic surveillance, (summarised in Table 1), can involve active or passive engagement with participants, whether it is performed at the individual or clinic level, or being healthcare or community-based. For instance, non-participatory passive approaches to syndromic surveillance include monitoring Google search terms [3, 4], or Tweets [5, 6], making use of large pools of data not specifically collected from participants for syndromic surveillance, but which can be analysed for trends in disease-related keywords. More indirect measures of disease occurrence can also be used in passive surveillance, such as school or hospital staff absenteeism [7] and pharmaceutical sales [8]. The majority of syndromic surveillance takes place at the level of a healthcare centre, employing personnel trained to collate reports [8, 9]. Participatory surveillance is a recent development in syndromic surveillance, and refers in particular to “the regular, voluntary submission of syndromic, health-related information by the general public using computers or smartphones”-[10], although this approach can make use of any electronic system capable of transmitting messages, be they web interfaces [11] or mobile phones [12]. The active role of participants introduces greater scalability and flexibility, whilst enabling the collection of highly detailed individual-level reports such as symptom time series. Participatory syndromic surveillance using mobile phones has been attempted through the use of custom-built applications such as that used in the Fluphone Project [12], which offer the advantage of being able to present participants with a clear interface of symptoms to select. Although extremely useful for its participant user-friendliness, a disadvantage of this approach is that it may not be possible to deploy the application on older phones used more commonly in developing countries, restricting the potential pool of participants. Whilst not capable of offering the same convenient interface, the basic text messaging capability of a mobile phone can also be used for this purpose. However, as free text for such reports would be unwieldy and very difficult to use effectively, these reports need to be structured in a clearly defined and consistent way. One of the main aims of our study was to pilot a coding system for symptoms that could be reported via text messages.

**Table 1:** Comparison of a range of approaches to syndromic surveillance.

Surveillance approach	Included population	Participant requirements	Administrator requirements	Technology required	Scaling	Advantages	Disadvantages
**Face-to-face interview**	Recruited participants	Time to complete interview, possibly transport to venue.	Trained interviewers, scripts.	Pen and paper/tablet computer	Poor. Additional participants necessitate greater interviewing effort.	Thorough disease reports, miscommunications may be avoided. Participants may feel more engaged.	Trained interviewers necessary, high burden upon participants, longitudinal study impractical, and poor scaling.
**Participant-completed online form**	Recruited participants	Completing online form.	Development of online form, reminders for participants.	Internet-capable device.	Electronic contact can readily scale.	Can be completed when convenient, ensure standardised reports, and include images etc. Scalable and automatable.	Internet access essential, and relies upon participant commitment. Reliant upon non-specialist reporting of symptoms.
**Participant text message reporting**	Recruited participants	Sending symptom reports and learning necessary syntax for reports.	Managing text message gateway, message processing, and training participants.	Mobile phone and text message gateway.	Electronic contact can readily scale.	Mobile phone access widespread. Can be sent when convenient for participants. Scalable, and automatable.	Reliance upon participant commitment and correct formatting without aid. Mobile access essential, and non-specialist reporting of symptoms.
**Monitoring of pharmaceutical sales**	Individuals using sentinel pharmacies	None	Collation of records/reports.	Electronic sales records.	Good if records standardised and automatically uploaded. Otherwise poor.	Large sampling pool. Minimal sampling effort.	Medication possibly poor proxy to disease incidence—purchasing behaviour not always be directly related to current illness.
**Monitoring of sickness absenteeism (medical personnel, schools etc.)**	Population of monitored institutions	None	Collation of records/reports.	Electronic absence records.	Good if records standardised and automatically uploaded. Otherwise poor.	Large sampling pool. Minimal sampling effort.	Selected institutions may be poor representations of disease patterns in the wider population, typically shallow disease information.
**Monitoring of electronic health records**	Visitors to sentinel healthcare centres	None	Collation of records/reports.	Electronic medical reports.	Good if records standardised and automatically uploaded. Otherwise poor.	Reliable and detailed reports. Relatively little sampling effort.	Bias towards serious illness. Appointments may focus upon a single ailment.
**Social media screening**	Social media using population	None	Development of appropriate message filtering and analysis algorithms.	Internet-capable device.	Good if filtering criteria and algorithms remain suitable.	Large sampling pool, relatively little sampling effort, potential geolocation.	Messages not intended for disease surveillance—filtering essential. Low report clarity expected.

Syndromic surveillance can scale very effectively to low-resource contexts [2, 13], as evidenced by the growing number of practical implementations of syndromic surveillance in developing countries for the monitoring of both human [8, 9, 14, 15] and animal [16] health. These examples include an electronic step in their implementation, from mobile phone-based reporting systems to convenient web interfaces for data entry. Access to technology in the developing world is continually increasing [17], even in highly remote settings, and mobile phones in particular are emblematic of this trend, combining the functionality of a landline with utilities such as mobile banking and internet access, even to those in highly remote settings. Nevertheless, relatively few published examples of the implementation of this technology exist, presumably in part due to variable levels of mobile phone ownership and operational challenges such as phone charging, language differences and reimbursement. For this reason, we sought to implement such an approach within a remote community in Western Uganda, where paper-based syndromic surveillance has been carried out several times in the past, to evaluate the practicality of this approach.

Uganda’s relationship with mobile phone technology is typical of that seen in many developing nations, with annually increasing levels of ownership indicated by rising numbers of mobile phone subscriptions; as of 2013, 47.7 mobile phone subscriptions exist for every 100 residents [18], although despite this substantial penetration of mobile phones, only 14.7% [17] of Ugandans use the internet. Alongside this growing access to technology, Uganda remains a nation where infectious disease is a significant problem, with an adult HIV prevalence of 7.2%[19], and malaria endemic throughout the country, alongside various other health threats including typhoid, trypanosomiasis and schistosomiasis [20]. Furthermore, Uganda was the location of several recent outbreaks of Ebola virus disease [21–23], highlighting the importance of early detection of emerging diseases and outbreaks in Uganda for global public health. With this combination of relatively high mobile phone availability, substantial disease burden and limited resources for disease surveillance, Uganda offers a highly suitable locale for assessing the implementation of mobile phone-based surveillance, and in particular one which takes advantage of community mobile phone ownership within a participatory epidemiology framework. However, just as with past syndromic surveillance performed at this site, our approach focused on providing high-resolution detail of patterns of disease within the human population, rather than outbreak detection. Unlike past paper-based surveys, use of a mobile phone-based approach enables far more efficient distributed collection of disease reports, offering the potential to collect detailed and precisely temporally situated reports. As such, although designed predominantly for methodological investigation, we sought to implement a study design optimised for high spatiotemporal resolution rather than coverage, concerned with detailing patterns of community disease rather than explicit outbreak detection, with this information providing a backdrop against which to detect anomalies.

We implemented syndromic surveillance in a rural community adjacent to Kibale National Park (hereafter Kibale) in Western Uganda, which consists of a mosaic of human settlements, tea plantations and forest fragments. The Kibale Ecohealth Project [24] has performed research within both the national park [25] and the communities around it [26], focusing upon a “one-health” perspective with regards to infectious disease. The situation around Kibale is different from that in West Africa, in that there is minimal bushmeat hunting in a context where primate contact is nonetheless commonplace due to raiding of crops by primates, and incidental contacts owing to human use of the forest [27]. Syndromic surveillance has previously been performed in the area, using paper surveys to gather symptom reports of current, and retrospective bouts of illness, creating a substantial pool of data pertaining to individual bouts of illness, but in which the asynchronous sampling of households renders time series analysis highly challenging, and reliance upon retrospective recall potentially introduces problems such as recall bias [28]. We implemented an alternative, mobile phone-based approach, to both gather a dataset which would avoid these limitations, and to investigate the viability of using such a method within this context. To assess the latter, we focused upon participant reporting behaviour, with particular attention paid to correct report formatting, and report frequency as indicators of comprehension and commitment, respectively. We also perform descriptive analyses upon the consequent dataset, with particular focus upon differences in illness duration and whether people other than the reporter being sick in the household is associated with the presence of certain symptoms, providing an indication of the association of these symptoms with different types of illness. Our experience illustrates both the potential for this approach for syndromic surveillance on a larger scale, and in other developing world settings, whilst also illuminating its present shortcomings.

## Methods

### Recruitment

A total of eighty participants were recruited in pairs from two communities adjacent to Kibale, referred to as “Bugembe” and “Kamakune”, with twenty households recruited from each of these. These communities, in effect, encompass a number of villages near particular fragments of forest that bear these names. Pairs of participants consisted of the head of household and a randomly selected household member. Participants were recruited using a randomly shuffled list of individuals who had participated in previous Kibale EcoHealth Project studies. This random process was influenced by the presence of participants at home during the one-week recruitment period, although households were also revisited if unavailable when initially visited. It was further influenced by field assistant knowledge from both prior surveys and their presence within the community as to which households had since moved, or owned mobile phones and thus were worthwhile to visit. This latter insight was relatively rarely used during this decision-making process, excluding approximately 5 households in total. The study was first explained to the head of household by a pair of field assistants in the local language, Rutooro, before written consent was sought. Participants were offered reimbursement for all messages sent, free phone charging arranged through local vendors, and a large bar of soap (a valued household commodity) at the end of the study. Participants were also offered mobile phone credit in advance of the study, with the amount provided to be subtracted from the total reimbursement at the end of the study. After the head of household recruitment, a second individual was selected from the household, based upon which household member’s date of birth had most recently passed. Participation included household members not present at the time, in which case the household would be revisited when convenient, and minors (individuals under the age of 18), from whom assent would be sought if they were capable of understanding and signing for themselves, alongside written consent from a parent or guardian. Ethical approval was granted from the Department of Geography, University of Cambridge, and the study was approved by both the Uganda Wildlife Authority and Uganda National Council of Science and Technology. A project determination was approved by the Human Subjects Office at CDC for participation in the study. As part of the consent and assent forms, participants were also asked to provide their age, sex, current household size, and mobile phone number, as demographic and identifying details.

### Procedure for participants

**Fig 1:**
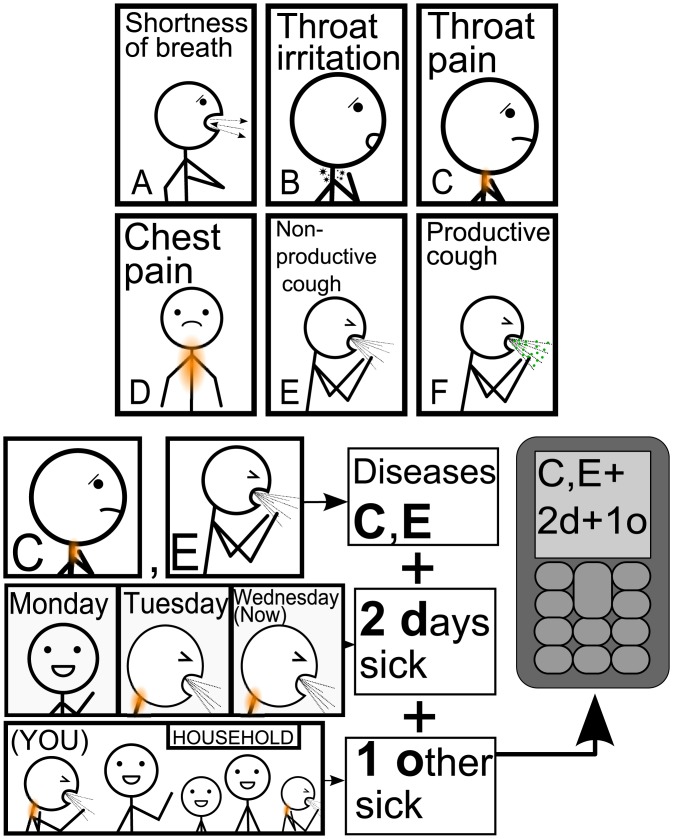
Sample of symptom codes, and pictogram explaining report structure. Depicted are a small subset of symptom codes, which includes febrile and gastrointestinal symptoms alongside some of the respiratory symptoms. Message structure consisted of alphabetical symptom codes, the number of days sick, and the number of other household members with the same illness. The first of these report elements is for the reporting of any current illness, number of days sick is intended to facilitate the distinction of different ‘bouts’ of illness, and the reporting of other household members sick with the same illness provides both an indication of household-clustering of illness, and allows for the testing of report consistency between household members.

Participants were asked to send text messages to a Short Message Service (SMS, or ‘text message’) gateway set up at the Makerere University Biological Field Station, consisting of a Raspberry Pi connected to a 3G modem, with the handling of text messages carried out with bash scripts and the Gammu package [29]. The study was initially planned to run over the course of 4 weeks, although this was later extended to add one additional week of reporting, to compensate for a high frequency of misunderstandings during the first week. Participants received a single reminder text message at synchronous 3-day intervals at 09:00 Eastern Africa Time, asking participants in Rutooro to send a symptom report. The protocol for reporting symptoms was explained with a combination of a ‘bookmark-sized’ laminated sheet given to each participant (see S1 Fig), which provided symptom codes via both pictograms and symptom translations from English into Rutooro, and basic message structure with a pictorial example, alongside an example sheet which provided additional examples of message structure. A subset of the 24 symptom images are shown in Fig 1, along with a pictorial example of report formatting which was provided to participants. Symptom codes were selected from past syndromic surveillance in the area, which identified common ailments witnessed and experienced. A small number of modifications were made to clarify certain symptom categories, and to add additional symptoms. In phone-owning households, the head of household typically owned a mobile phone, whilst others in the household were far less likely to own one, leading to the sharing of phones for reporting in these cases. In these situations, participants were asked to preface their messages with a 1 if they were the head of household, or a 2 if not.

### Procedure for field coordinator and assistants

During the first week of the study, all participants were revisited by a field assistant with the field coordinator to check for any initial questions or issues. Over the study these visits became less frequent, focusing only on those who sent incorrectly formatted messages, and who had failed to send their last two reports, such that individuals who consistently sent correctly formatted text messages went entirely unvisited until the end of the study.

### Defining and analysing bouts of illness

We divided the time series of symptom reports into continuous periods of illness, referred to as “bouts”. In principle, the splitting of report time series can be carried out using a range of criteria: splitting reports if a symptom ceases to be consecutively reported, a period of health is reported, or if the next report of illness is stated to be very short in duration (indicating it is a new bout of illness). The latter two criteria were used, ensuring that periods of illness in which symptoms changed were not split into multiple bouts, whilst splitting across good health and short-duration illness. It is important to note that this is a relatively crude approach to distinguishing between bouts of illness, as it takes no account of recurrent clusters of symptoms which may provide better and more context-relevant distinction between bouts, or of changes in symptoms during bouts. Given the disproportionately high number of single-timeslot length bouts, the median was used in this comparison to lessen skew towards these low values, whilst the mean was used for the less skewed distribution of proportions of reported others reported to be sick with the same illness.

## Results

### Overview of reporting behaviour

**Fig 2:**
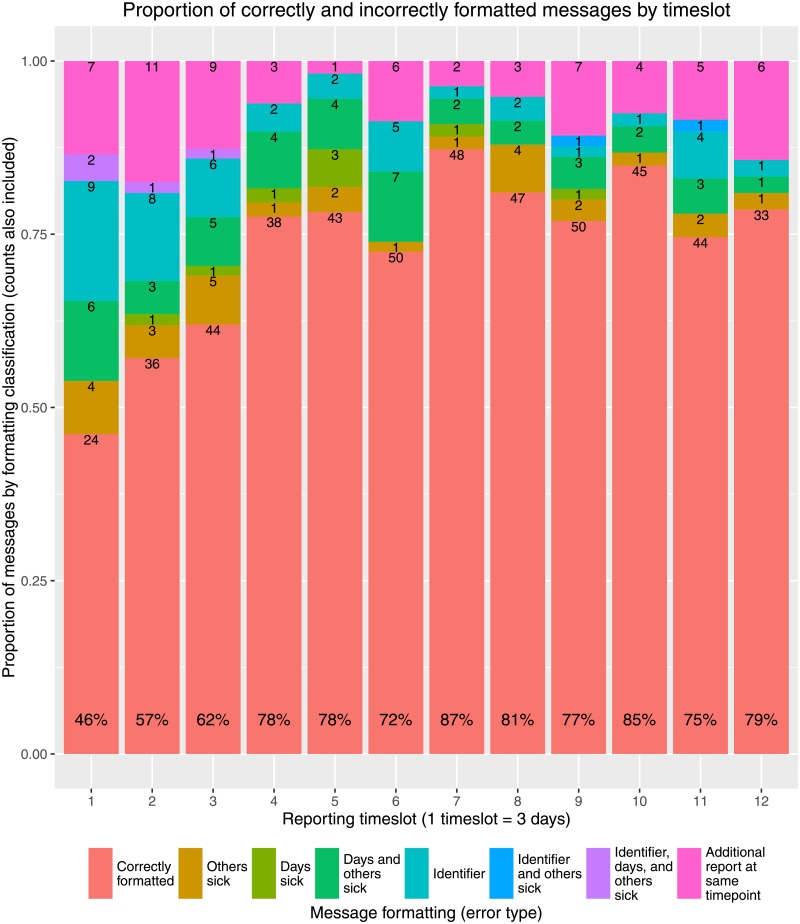
Overview of participant reporting behaviour. Basic breakdown of message formatting at each timestep, with total percentage correct at the base of each bar.

**Fig 3:**
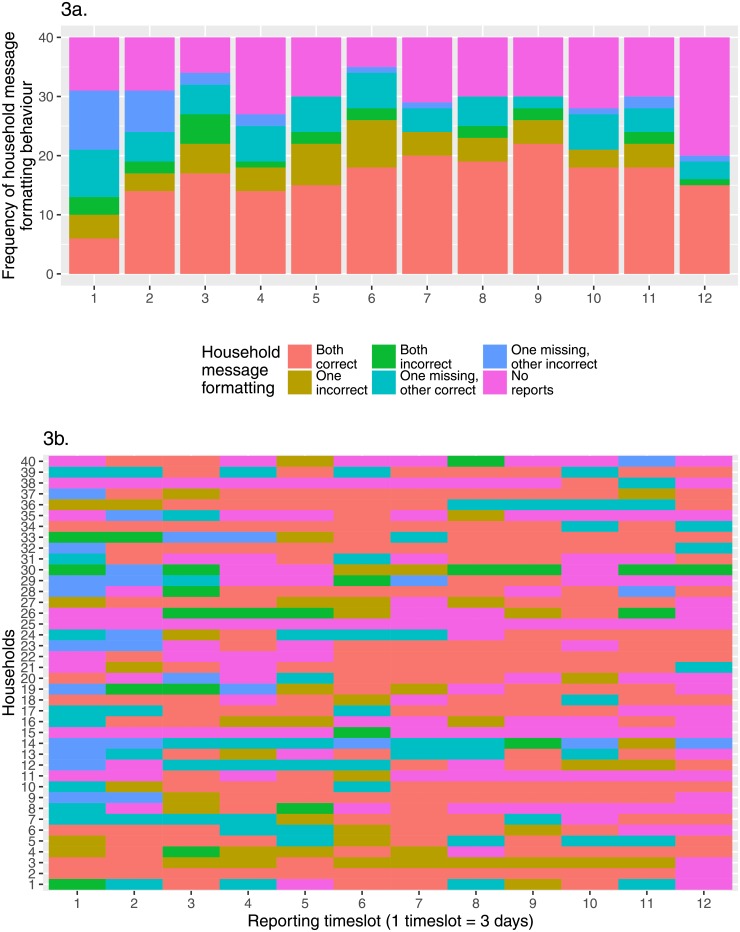
Household-level reporting behaviour. Categorisation of households based upon message-sending behaviour. For each 3-day reporting timeslot, the overall proportions of different reporting behaviours (**3a**) and the reporting behaviour of each household (**3b**).

**Fig 4:**
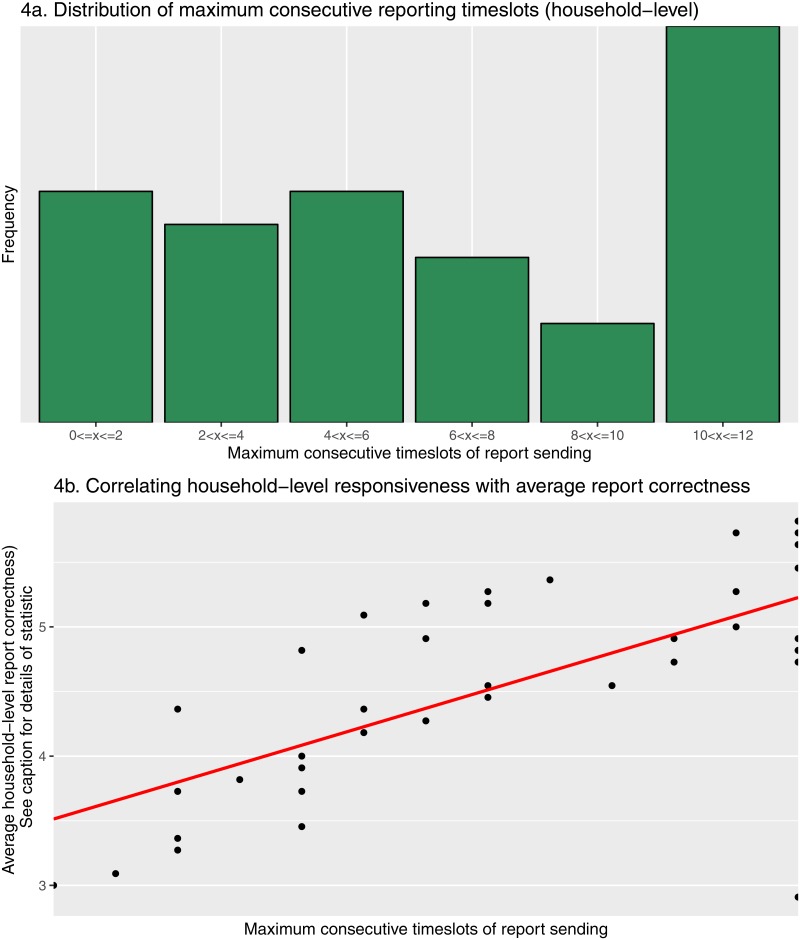
Household-level reporting consistency. Descriptive statistics pertaining to household responsiveness. Consecutive reporting is determined on the basis of receiving at least one report at the household-level, correct or incorrect (**4a**). Also considered is the relationship between overall reporting consistency and average report accuracy (**4b**). The statistic used for household-level correctness is derived from the 12 timeslot average of coding household report behaviour as follows: No reports = 1; one report, incorrect = 2; one report, correct = 3; two incorrect = 4; two reports, one incorrect = 5; two reports, correct = 6.

Fig 2 illustrates the number and proportion of reports by different error types, examples of which are characterised in Table 2. Errors were determined purely on the basis of message formatting, without external validation. As expected, the first reporting date had the lowest proportion of entirely correct reports, as participants made their first attempt at independently sending reports. Report correctness subsequently increased, with a mean of 75.2% (sd = 9.43%) of symptom reports correctly formatted between the second and eleventh reporting timeslot. The lower than expected number of reports in the final timeslot likely reflects the end of participant reimbursement, such that many individuals may have considered the study to be at its end. Furthermore, the high proportion of correctly formatted messages may indicate that the participants sending reports at this point were those particularly committed to, and understanding of, the study. The most frequently seen errors over the study period were the sending of duplicate reports (mean 34.9% (sd = 14.7%) of errors), followed by incorrectly reported days and others sick (mean 23.4% (sd = 8.83%) of errors) and the absence of identifiers for shared household reports (mean 19.5% (sd = 7.92%) of errors), the latter two of which declined in frequency over the course of the study. At the household level, after the majority of households sent at least one incorrect report on the first reporting date, rapid improvement in the number of households for which both reports were correct was seen until the fourth reporting timeslot. Thereafter increase in correct reporting occurred more slowly, before plateauing between timeslot 7 and 11 (Fig 3a). Interestingly the frequency of receiving one correct and one incorrect report, or one missing and the other correct, was fairly consistent across the study, whilst that of one missing with the other incorrect diminished. There was a relatively clear division between households that consistently sent reports over the course of the study, occasionally with minor issues, such as households 32 and 34, and households that proved less responsive throughout the study, such as households 15, 25, and 48 (Fig 3b). This is further detailed in Fig 4a, the most responsive households (> 10 consecutive timeslots of report sending) can be clearly distinguished from those nearer the mean of 6.98 (sd = 3.87) consecutive reports, and a notable absence of tailing off in frequency of those below this point, particularly notable for the least responsive. In Fig 4b a clear correlation can be seen between consistent report sending and average household report correctness, with the least consistent households those most likely to send incorrect reports, and the inverse true of the most consistent. This analysis is partially confounded by households in the lowest ‘correctness’ category (those sending no reports) also likely those less consistently sending reports. However, that this correlation can be seen across the wide range of maximum consecutive timeslots considered suggests this effect is not simply an artifact, with low ‘correctness’ scoring a natural product of low consistency alone.

**Table 2:** Examples of syntax errors in text message reports, where ‘?’ indicates a missing report element, and ‘NA’ an unneeded report element. Note ‘problem with identifier and days’ not included, due to no examples of this occuring.

Error type	Original text	Identifier	Symptoms	Days sick	Others sick
Correctly formatted	H,i+3w+1o	NA	H,I	3	1
Problem with identifier	X,o 2d	?	X	NA	NA
Problem with days sick	2+A,D,F,M,N,S+4O	2	A,D,F,M,N,S	?	4
Problem with others sick	F,K,R+3d	NA	F,K,R	3	?
Problem with days and others sick	V+2d.	NA	V	2	?
Problem with identifier and others sick	2+F	2	F	?	?
Problem with identifier, days and others sick	C	?	C	?	?

Only one household sought to leave the study, and did so within the first week of reporting, attributable to a chronic medical condition experienced by the head of the household, who owned its only mobile phone. This rendered it difficult for them to regularly obtain phone charging and phone credit.

### Overview of symptom reports

**Fig 5:**
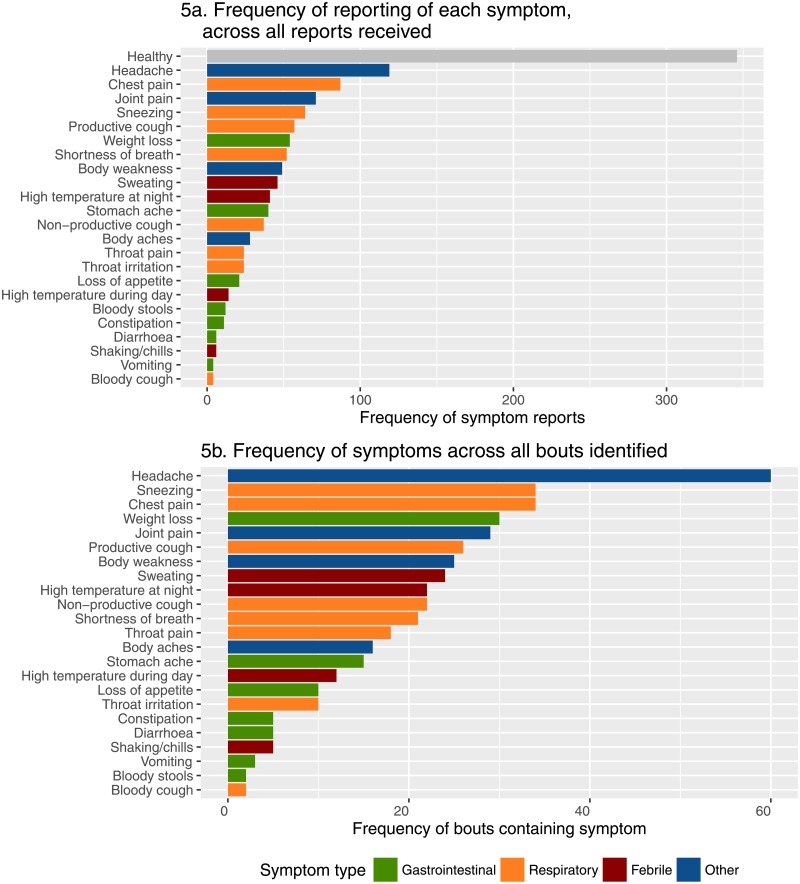
Number of mentions of each symptom across all reports, coloured by symptom type (5a). Frequency of distinct bouts containing at least one mention of a given symptom (**5b**).

**Fig 6:**
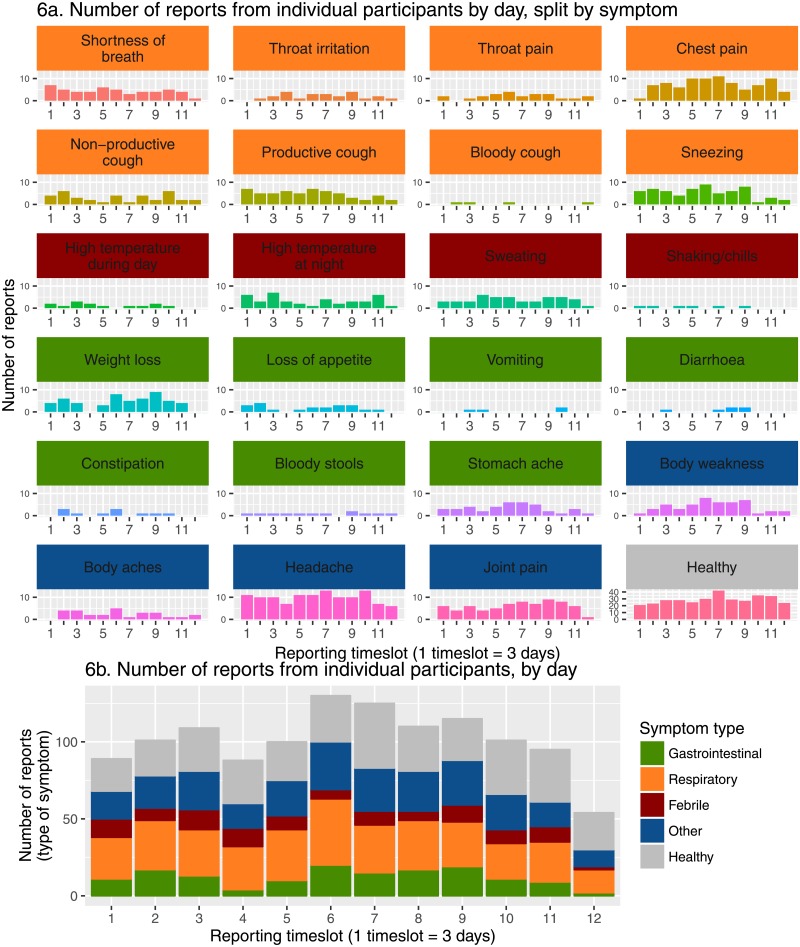
Symptom report totals by timeslot. Mentions of individual symptoms in reports by timeslot(**6a**), total symptom reports of a given type by timeslot (**6b**).

The most frequently reported symptom was good health (28.4% of total reports), however a large number of reports contained mentions of illness (Fig 5a). Most abundant were reports of headache (9.78%), often in isolation, but also alongside other symptoms, meanwhile the most rarely reported symptoms were bloody cough and vomiting (both 0.339%). Overall, respiratory symptoms such as sneezing and chest pain were most commonly reported, followed by febrile symptoms, whilst gastrointestinal symptoms were reported relatively rarely. While these results are influenced by relative differences in the numbers of symptoms within broad categories, it nonetheless provides some indication of the types of illnesses most frequently reported. However, there is a potential bias to detect symptoms associated with longer periods of illness. Fig 6a shows in further detail which symptoms were most frequently reported, and how this varied over the surveillance period. As might be expected for this relatively short five weeks period of surveillance, no major apparent trends or fluctuations in the reporting of different symptoms were evident (Fig 6b).

### Distinguishing periods of illness

Assessment of measures of the durations of illness and the reported numbers of “others sick” in households is slightly more complex than that of symptoms alone, as it necessitates the splitting of the symptom time series into discrete periods of illness, hereafter referred to as “bouts”. Fig 5b depicts the frequency of bouts containing a particular symptom; the overall pattern is similar to that in Fig 5a, however symptoms associated with longer bout durations such as shortness of breath were less frequent. Nonetheless, headache remained the most frequent symptom observed in bouts (14.0%), followed by chest pain and sneezing (both 7.91%).

**Fig 7:**
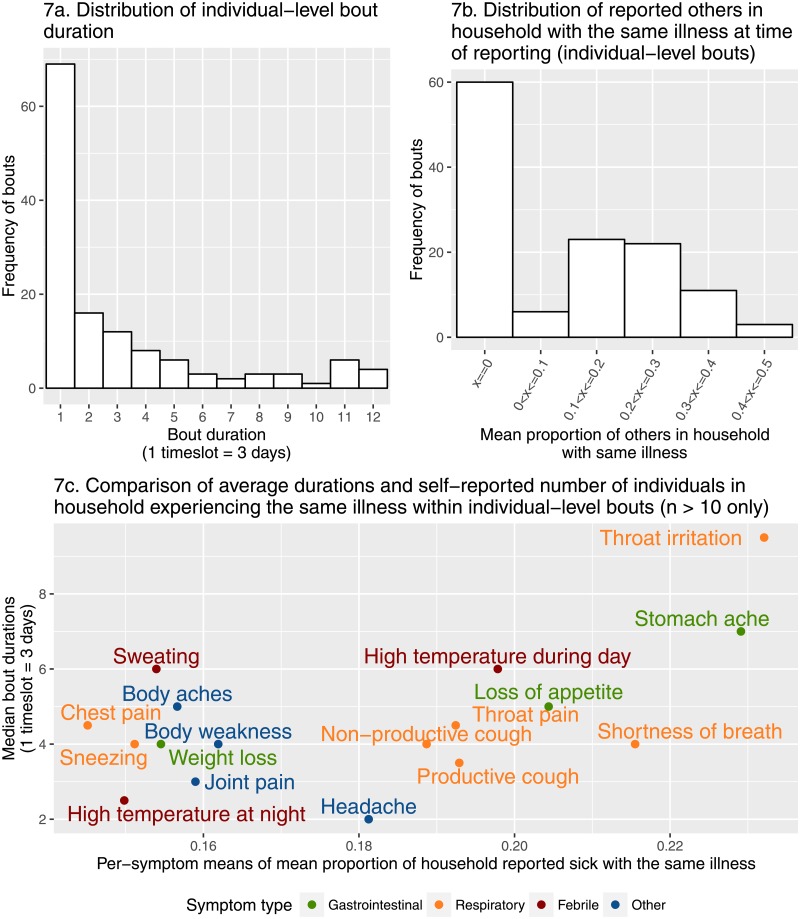
Between-bout comparisons. Distributions of bout duration (**7a**) and the number of others reported to be sick with the same illness (**7b**) during bouts of illness. Also, comparison of the means of summary statistics obtained by aggregating all bouts containing at least one mention of a given symptom—median bout duration, and mean proportion of others sick (**7c**). This proportion was calculated using the number of individuals living in the household, which was recorded as part of the consent/assent process, and the distribution of which can be seen in S2 Fig.

Most illnesses lasted for a single reporting timeslot (i.e. three days or less), although the distribution of bout length had a long tail (Fig 7a). The majority of bouts included reports of at least one other case, although reports of more than half of household members being affected by illness did not occur (Fig 7b). When we combined information on both the reported durations and others sick in the same household, bouts containing symptoms such as throat irritation, shortness of breath and stomach ache tended to involve more members of the households in which the bouts occurred (23.2% (sd = 14.9%), 21.6% (sd = 17.6) and 22.9% (sd = 21.4%) respectively) (Fig 7c). Bouts involving symptoms such as chest pain and high temperatures at night (a symptom, along with high temperature during day, aiming to capture different forms of fever) tended to affect a smaller proportion of household members (means 14.5% (sd = 15.5) and 15.0% (sd = 14.6%) respectively). Bouts containing at least one report of headache or high temperature at night tended to be the shortest (median 2.00 (IQR = 4.00) and 2.50 (IQR = 7) timeslots), and those containing throat irritation the longest (median 9.50 (IQR = 4.5) timeslots).

In some cases within-household bouts sharing symptoms appeared to show disagreement regarding number of ‘others sick’ even in the presence of shared symptoms. Dealing with this disagreement is challenging, as participants were explicitly asked to note others they deemed to be experiencing the same illness, rather than simply others who might share a symptom. Within our sample, there were no instances in which more than 40% of symptoms were shared between simultaneous within-household bouts such that without more detailed syndromic assessment we considered it unreasonable to deem any of these reports incorrect, and modify them accordingly.

## Discussion

Disease surveillance is a complex challenge, and large-scale implementations can be highly resource-intensive. With increasing access to telecommunications and the internet, an electronic approach holds the potential not only to provide a highly resource-efficient means of disease surveillance, but also to allow researchers to obtain detailed data, such as individual symptom time series, which otherwise would be prohibitively resource-intensive to gather. Our platform employs text messaging, and hence can be deployed in many areas, including developing countries, and our coding system for symptoms and their duration covers a greater breadth of symptoms than prior studies. Our setup requires only a local mobile telephone number, and means of storing received text messages; we employed a Raspberry Pi server in the local area, although in other settings, it may be possible to transmit these messages to a cloud-based server, removing the need for local hardware. The main barrier to the success of our approach is the extent to which participants can correctly use the symptom coding system.

Participants who sent incorrect messages received detailed verbal explanations of how to fix their errors, and a reduction was seen in the frequency of ‘both incorrect’ and ‘one missing, other incorrect’ patterns of reporting. Whilst this indicates the learning of correct formatting in some cases, the overall proportion of ‘both correct’ formatting plateaued over time. Indeed, there existed a core of participants for whom the explanation provided was adequate, alongside a number of participants for whom it was not, even after verbal follow-up. Along with illustrating a division in comprehension, this divide could indicate a split between households that were more or less engaged and motivated to both participate and ensure their reports were correct. By its very nature, participatory surveillance is heavily reliant upon participants making active contributions, and individuals more enthusiastic about participating might be expected to report more accurately than those who were more disinterested. To improve participant comprehension, a greater number of written examples could be provided, making it easier for participants to verify that they were sending correctly formatted reports. A possible further way to provide motivation would be to promote the role of individuals and households more consistent and correct in their reporting to support less proficient neighbours. This could involve greater community engagement to facilitate stronger background reinforcement of reporting behaviour, or a more specific programme of pairing more proficient households with those experiencing difficulties. Both of these strategies could serve not only to directly improve reporting, but also to develop a greater sense of engagement and ownership of the surveillance work, which would be essential in longer term or unsupervised implementations.

Alternatively, it is useful to reframe this division in the context of sentinel surveillance, wherein disease surveillance is directed at a subset of a population. This subset can be designed to be population-representative, or those groups deemed at-risk, which is particularly relevant to disease emergence. Although the case study reported here does not readily fit within either category, particularly with limited number of participants, the division between high and low engagement households could suit a typical sentinel approach, with particularly competent reporters focused upon, and those less competent removed and replaced. This replacement could ensure more consistent reporting, particularly within the context of long-term implementation, but at the expense of granularity. Whilst a sentinel approach would be best suited to the detection of unusual patterns of disease, and particularly outbreaks, selecting for high commitment could result in a more biased sample for household-level monitoring of disease and result in unmonitored ‘gaps’ within the population studied. Our data suggest the fundamental requirements for both approaches, including when implemented using mobile phones, exist in this context, with the preferable option determined by the desire for more consistent, or comprehensive reporting. Whilst an approach rooted in sentinel surveillance might better suit a public health focus, the spatiotemporal detail provided by our approach would be of greater value for detailed research upon household-level patterns of disease and transmission, and if implemented at sufficient scale within a single, or multiple small communities, potentially valuable from a public health perspective.

An important potential source of bias is access to mobile phones. We sought to avoid this bias by allowing single-phone households to use a shared device, given that the majority of households possess at least one mobile phone. This approach also helps to account for possible differences in mobile phone ownership based upon age, as younger household members who are generally more familiar with mobile phone text messaging can guide and support those less familiar. Still, it is possible that some older, or poorer households were excluded due to lack of mobile phone access. This is particularly notable as such households might be expected to experience a disproportionately higher burden of disease owing to factors such as reduced access to healthcare due to financial limitations. Such biases are a fundamental limitation of a mobile phone-based approach; however with mobile phones increasingly tailored for the developing market in both price and specification, ownership would only be expected to increase. As such, the effect of this bias should be slight, and in future even smaller. Meanwhile the reliance of recruitment upon previously surveyed households might have biased the sample towards more compliant households overall (owing to their past participation), and towards households better known to the field assistants owing to the use of participant insights during recruitment. Whilst this first bias cannot be excluded, experience from numerous past surveys would be expected to ensure field assistants were impartial in their insights, and as very few households were omitted based upon them, any bias from this process was small.

A further source of bias could have been produced by the withdrawal of participants from the study, with such departures having the potential to bias the sample overall towards a particular ‘type’ of participant. Fortunately however, only a single participating household sought to leave the study, suggesting that it was largely the exceptional circumstances of the head of household which resulted in their departure. In response, a new household was recruited, one visited during the recruitment period of the study, but which was empty at that time. Whilst this flexibility was not initially an aspect of the study, it is clear that participant replacement to provide a cohort of constant size in the event of participant withdrawal, or potentially continued non-reporting, would be a necessity if this method of syndromic surveillance were to be enacted over an extended period of time.

To improve reporting, whilst allowing the widest range of mobile phones, an automated interactive approach could be taken. Our current platform involves mainly one-way communication between the participant and the server, with text messages being sent at fixed intervals to participants only to remind them to report. The server could validate incoming text messages, and reply to individuals who have sent incorrectly formatted messages immediately. Another potential approach—although not available in all settings—is to employ an interactive voice response (IVR) system, which, much like telephone banking, prompts individuals for specific answers. Like the two-way text messaging approach, IVR has a wide reach in terms of mobile phones, but would be more time consuming and expensive to deploy and validate.

Several of the most commonly reported symptoms were related to respiratory illness. This result is not unexpected, both due to the range of possible aetiologies (respiratory tract infection, allergies, exposure to irritants), and obvious and well-defined nature of many respiratory symptoms. Symptoms of febrile illness were comparatively less common, despite malaria being an endemic illness throughout Uganda. However, Kabarole (the district containing the study communities) does experience a lower prevalence of malaria than much of Uganda [30], owing in part to its relatively high altitude (Kibale ranges between 1,390-1,625m above sea level [31]). We caution, however, that these results likely also reflect seasonality of disease and the short duration of this study. Participant subjectivity could also lead to reduced reports of certain symptoms considered “normal” by participants (e.g. mild fever), or of symptoms which participants may feel uncomfortable reporting (e.g. gastrointestinal illness). When participants were introduced to the study, efforts were made to avoid these potential biases, with participants asked to report all symptoms experienced, and clearly informed of the confidentiality of symptom reports. Whilst no participants expressed concerns about the reporting of any symptoms in particular, this possibility cannot be excluded.

The reports of others sick and days sick accompanying the reports of symptoms lend a rare depth to the disease data gathered. Based upon the bouts derived from the dataset, most reported illnesses were relatively short in duration and tended to involve relatively few other household members. Bouts associated with the lowest mean proportion of others sick were those involving chest pain. Whilst listed as a respiratory symptom, chest pain offers considerable scope for variation in interpretation, given that it can be used to describe any form of pain in the torso, for instance that stemming from heartburn. Bouts containing symptoms such as throat irritation, shortness of breath, and stomach ache displayed the highest mean proportions of others sick, and a range of mean durations. Two of these symptoms (throat irritation and shortness of breath) are categorised as respiratory symptoms, and may indicate that at least some of these bouts relate to an infectious aetiology, which could explain why bouts containing these symptoms tend to affect a greater proportion of household members. Meanwhile stomach ache could be interpreted as being indicative of gastrointestinal disease associated with a shared contaminated food source, or potentially infectious aetiology. It is of note that on the scale of mean proportion of household reported sick, the next symptoms are a collection of numerous respiratory symptoms alongside a sole gastrointestinal and febrile symptom. The tendency towards higher “others sick” for these respiratory symptoms could again be interpreted as indicating some bouts influenced by shared environmental exposure or an infectious aetiology. Appetite loss is similarly congruous when considered alongside reports of stomach ache, with both symptoms potentially pertaining to shared contamination or infection. The sole febrile symptom here meanwhile, high temperature during the day, could be interpreted as being indicative of some measure of fever, which could again be indicative of an infectious aetiology.

The analysis of symptoms, durations and others sick performed here was mostly descriptive, and treated symptoms as individual entities. However, in reality, a single infectious disease may cause an array of symptoms, each of which may manifest at different stages of infection. More sophisticated statistical techniques are needed to extract groups of symptoms (“syndromes”) from the available dataset, and to distinguish bouts in a more context-sensitive way. The high dimension of the data (24 symptoms), the composition of the data in terms of binary (symptoms present or not) and count (duration of symptoms in days) components, the repeated measurements over time, of individuals from the same household, and the potential for higher-level clustering in space and/or time between symptoms in different households all present significant challenges to the development of statistical models that can extract the most information out of the data generated by our platform. As previously noted, such syndromic clustering would also aid in identifying possible errors in reports. Notably, disagreements in the numbers of ‘others sick’ were seen despite of the presence of some shared symptoms. Considering bouts only at the symptom level renders it difficult to distinguish ‘truly’ different illnesses which should not be counted from those which could be classified as the same. Also, we emphasise that our study was not designed expressly to assess the accuracy of symptom reporting, but rather the feasibility of the overall approach for tracking changes over time.

While this study mostly used an automated system, field assistants played a vital role—to introduce the study and train participants in correct reporting of symptoms. However, as the bulk of recruitment and training occurred at the beginning of the study, the need for human resources was highest in the first week of study. Following that, the number of field assistants was reduced to deal with those who consistently report incorrectly, and to recruit additional participants to compensate for study drop-out. Further software development, for example offering a web-based system to review frequency and correctness of reports, would help field assistants administer the study over a wider geographic area. The greatest challenge to scalability thereafter lays in the time required to both recruit, and thereafter manage self-reporting, and participant reimbursement. The recruitment of 80 individuals required roughly a week owing to time necessary for travel and thorough explanation (≈ 1 hour). However with more than a single pair of field assistants, the efficiency of this step could be enhanced significantly. Similarly, the majority of participants were visited at least once following initial recruitment, however these visits were typically short (≤ 15 minutes) as they concerned minor clarifications or prompting. At a large scale, even these short visits could become unmanageable, as such a greater focus might need to be placed upon text prompts, or even some measure of automated error detection based upon the standard syntax used.

Reimbursement is a crucial aspect of this case study, aiming to remove all but time expense on behalf of participants, and in doing so aiding both recruitment and ongoing participant retention, however in this case study it was managed entirely manually which, whilst effective, would ideally be managed automatically. Fortunately, this too could be automated, with various mobile networks capable of processing credit transfers automatically through text messages alone, enabling functionality such as immediate reimbursement upon reporting. Free phone charging can be more readily automated, as it was in this study, with credit arrangements made with charging operators themselves requiring infrequent visits to settle outstanding balances. With relatively simple changes to help participants regularly send correct reports, and to provide greater scope for field assistant administration, the benefits of the scalability of this participatory electronic approach can begin to come into play. Further extensions to the software also have the potential to significantly reduce field assistant workload, again taking advantage of the scalability of the electronic approach to maximise the efficiency of data collection, rendering this a concept which could reasonably be deployed at a large scale. Whilst doing so would include a great many challenges, as outlined, it would also have the potential to gather datasets unique in their depth. Our platform, employing active surveillance of a wide range of symptoms, avoids many issues associated with approaches that mine data streams for key words, and has the potential to generate rich datasets concerning spatiotemporal patterns of disease.

## Supporting Information

S1 Fig The symptom reporting information sheet. This is the information provided to participants, in the form of a laminated ‘bookmark’, which comprises of pictograms of 24 different symptoms, with the names of the symptoms in Rutooro, the local language. The right hand side depicts the coding system used in the text messages.
(PDF)

S2 Fig Histogram of participating household sizes. Used for the calculation of proportion of ‘others-sick’ as in Fig 7.
(EPS)
