# Supplementary figures and images for: Assessing Commitment and Reporting Fidelity to a Text Message-Based Participatory Surveillance in Rural Western Uganda
Source: PLoS One. 2016 Jun 9;11(6):e0155971. doi: 10.1371/journal.pone.0155971 (PMC4900526; doi:10.1371/journal.pone.0155971)

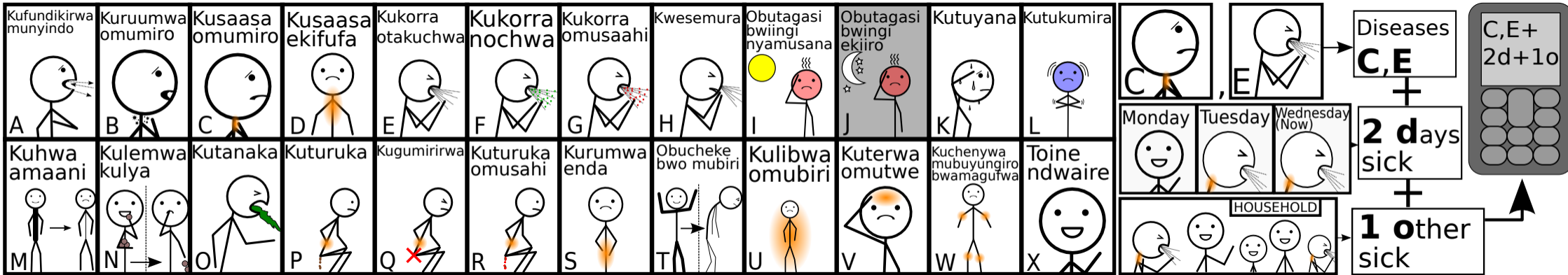

Supplement: S1 Fig — This is the information provided to participants, in the form of a laminated ‘bookmark’, which comprises of pictograms of 24 different symptoms, with the names of the symptoms in Rutooro, the local language. The right hand side depicts the coding system used in the text messages. (PDF) [file pone.0155971.s001.pdf]
